# Fast Detection of Nutrient Limitation in Macroalgae and Seagrass with Nutrient-Induced Fluorescence

**DOI:** 10.1371/journal.pone.0068834

**Published:** 2013-07-05

**Authors:** Joost den Haan, Jef Huisman, Friso Dekker, Jacomina L. ten Brinke, Amanda K. Ford, Jan van Ooijen, Fleur C. van Duyl, Mark J. A. Vermeij, Petra M. Visser

**Affiliations:** 1 Aquatic Microbiology, Institute for Biodiversity and Ecosystem Dynamics, University of Amsterdam, Amsterdam, The Netherlands; 2 Aquaculture and Fisheries Group, Wageningen University, Wageningen, The Netherlands; 3 Royal Netherlands Institute for Sea Research (NIOZ), Den Burg, Texel, The Netherlands; 4 CARMABI Foundation, Willemstad, Curaçao; University of Melbourne, Australia

## Abstract

**Background:**

Rapid determination of which nutrients limit the primary production of macroalgae and seagrasses is vital for understanding the impacts of eutrophication on marine and freshwater ecosystems. However, current methods to assess nutrient limitation are often cumbersome and time consuming. For phytoplankton, a rapid method has been described based on short-term changes in chlorophyll fluorescence upon nutrient addition, also known as Nutrient-Induced Fluorescence Transients (NIFTs). Thus far, though, the NIFT technique was not well suited for macroalgae and seagrasses.

**Methodology & Principal Findings:**

We developed a new experimental setup so that the NIFT technique can be used to assess nutrient limitation of benthic macroalgae and seagrasses. We first tested the applicability of the technique on sea lettuce (*Ulva lactuca)* cultured in the laboratory on nutrient-enriched medium without either nitrogen or phosphorus. Addition of the limiting nutrient resulted in a characteristic change in the fluorescence signal, whereas addition of non-limiting nutrients did not yield a response. Next, we applied the NIFT technique to field samples of the encrusting fan-leaf alga *Lobophora variegata*, one of the key algal species often involved in the degradation of coral reef ecosystems. The results pointed at co-limitation of *L. variegata* by phosphorus and nitrogen, although it responded more strongly to phosphate than to nitrate and ammonium addition. For turtle grass (*Thalassia testudinum*) we found the opposite result, with a stronger NIFT response to nitrate and ammonium than to phosphate.

**Conclusions & Significance:**

Our extension of the NIFT technique offers an easy and fast method (30–60 min per sample) to determine nutrient limitation of macroalgae and seagrasses. We successfully applied this technique to macroalgae on coral reef ecosystems and to seagrass in a tropical inner bay, and foresee wider application to other aquatic plants, and to other marine and freshwater ecosystems.

## Introduction

Eutrophication can lead to highly adverse changes in the structure and functioning of freshwater and marine ecosystems [1]–[3]. Enrichment with nitrogen (N) and phosphorus (P) often relieves primary producers from nutrient limitation, enhancing the productivity of micro- and macroalgae. This may result in reduced water clarity, development of harmful algal blooms, nighttime oxygen depletion, strong diel fluctuations in pH, and the smothering of coral reefs and other benthic communities [2], [4]–[6]. Therefore, a fast and easy method to identify which nutrients limit the primary production of micro- and macroalgae can be of considerable value to assess potential effects of future nutrient enrichments, and may help to increase the effectiveness of nutrient reduction programs in a wide variety of different water bodies.

Existing methods to assess nutrient limitation in macroalgae and aquatic plants are based on (1) analysis of ambient nutrient concentrations [7], [8], (2) element ratio analysis of algal tissue [8]–[10], and (3) nutrient enrichment assays [8], [11]–[14]. Analysis of ambient nutrient concentrations in the overlying water can be fast, but is not sufficiently informative to determine the nutrient status of benthic organisms. Element ratio analysis of algal tissue and nutrient enrichment assays may take considerable amounts of time to identify nutrient limitation in algae, often lasting several hours or days. Furthermore, especially in nutrient enrichment assays, the organisms are often studied under artificial conditions, possibly complicating the interpretation of results. Hence, there is a need for a fast and informative technique that can be easily applied *in situ*. For phytoplankton, such a method exists in the form of Nutrient-Induced Fluorescence Transient (NIFT) experiments, where nutrient limitation can be detected within minutes [15].

NIFT experiments are based on the principle that addition of limiting nutrients induces transient changes in chlorophyll *a* fluorescence, which can be detected with a Pulse Amplitude Modulation (PAM) fluorometer [15]–[20]. Enhanced uptake and assimilation of limiting nutrients increases the demand for ATP and/or reductants. This relieves pressure on the photosynthetic electron transport chain, which can alter non-photochemical quenching, the redox state of the plastoquinone pool, state transitions between photosystems I and II, and the relative importance of linear versus cyclic electron transport [21]. These changes affect the fluorescence signal since the processing of absorbed light energy by photochemistry, fluorescence and heat dissipation occurs in competition [22]. Hence, a transient change in fluorescence upon nutrient addition provides direct evidence for a change in algal nutrient status. When a non-limiting nutrient or distilled water is added to a phytoplankton culture, generally no change in fluorescence is observed [9].

Since the photosynthetic apparatus operates essentially in a similar way across all oxygen-producing phototrophic organisms, the NIFT technique should in principle be applicable not only to phytoplankton but also to macroalgae, seagrasses and other aquatic plants. However, a major obstacle for application of the NIFT technique to macroalgae and aquatic plants is that they cannot be homogeneously resuspended in a cuvette, which is standard procedure for microalgae [9], [15]. The leaf clips commonly used in PAM fluorometry with macroalgae and seagrasses are not suitable for NIFT studies, because they either cannot hold the sampled leaf at exactly the same position or they interfere with full access of the leaf to the nutrients added during a NIFT experiment. To address this issue, we developed a special set-up that we have called the PAM fluoroscope. This set-up uses a magnetic leaf clip that allows easy and even addition of a nutrient pulse, while keeping the sample in exactly the same position in front of the PAM sensor.

In this study, we tested the applicability of the NIFT technique to macroalgae and sea grasses. We first used laboratory-controlled conditions to ensure that sea lettuce (*Ulva lactuca*) became either N or P starved, and followed its fluorescence after re-supply of the limiting and non-limiting nutrient to assess its NIFT response. After successful testing of the method, we collected samples of the macroalga *Lobophora variegata* from a degraded and less degraded coral reef, and assessed by which nutrient it was limited. Similar experiments were conducted with the seagrass *Thalassia testudinum*, growing in a nearby bay.

## Materials and Methods

### Research Sites

**Figure 1:**
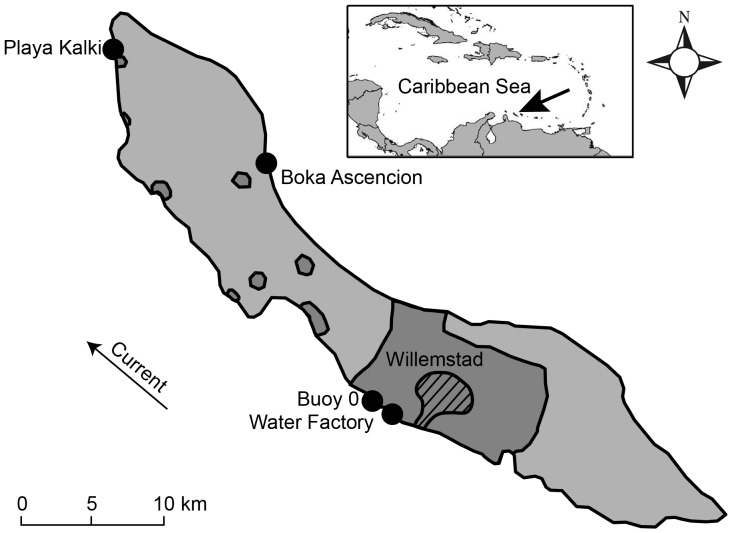
Map of Curaçao. Map with research sites Playa Kalki, Boka Ascencion, Buoy 0, and Water Factory on the island of Curaçao, Southern Caribbean (12°10′N, 68°58′W). Shading indicates urban areas (dark grey zones) and the commercial harbour (striped area).

This study was conducted on the island of Curaçao, Southern Caribbean, at research sites ‘Buoy 0’ (12°7′N, 68°58′W), ‘Playa Kalki’ (12°22′N, 69°9′W), ‘Water Factory’ (12°6′N, 68°56′W), and ‘Boka Ascencion’ (12°16′N, 69°3′W) (Fig. 1). Buoy 0 and Playa Kalki are both coral reef ecosystems. However, Buoy 0 is a more degraded reef, with a lower cover by hard corals and higher cover by macroalgae and turf algae than Playa Kalki. The site Water Factory is characterized by large beds of sea lettuce in the intertidal zone. Boka Ascencion is a shallow inner bay with large beds of turtle grass. Permission to conduct our studies was provided by the Ministry of Health, Environment and Nature (GMN) of the government of Curaçao through their permit (#48584) to the Caribbean Marine Biological Institute (CARMABI) at Willemstad.

### Laboratory Incubation of *Ulva lactuca*

Samples (∼2 cm^2^) of leaves of sea lettuce (*Ulva lactuca* Linnaeus) were manually collected from the intertidal zone at the Water Factory. The sampled leaves were transported to the laboratory facilities of CARMABI, where all NIFT experiments were conducted. During transport from reef to laboratory, samples were kept at a temperature of 27–29°C and shaded using a small cool box with seawater collected at the sampling location.

To test the presence of a NIFT response under controlled laboratory conditions, the collected *U. lactuca* leaves were starved of either N or P for three weeks. Samples were incubated in 300 ml glass incubators containing filtered seawater (Whatman cellulose acetate membrane filters, pore size 0.22 µm, Ø 25 mm) collected from surface water at Buoy 0. The nutrient concentrations in this seawater were 0.25 µM NO_3_^−^, 0.90 µM NH_4_^+^, and 0.07 µM PO_4_^3−^. Each sample received additional FeCl_3_ (0.16 µM) to ensure that iron did not become a limiting factor. To prepare P-limited medium, NO_3_^−^ and NH_4_^+^ were added to the filtered seawater at final concentrations of 5.1 µM and 18.6 µM, respectively. To prepare N-limited medium, PO_4_^3−^ was added at a final concentration of 1.4 µM.

Glass incubators with P-limited and N-limited medium were placed in triplicate inside an aquarium, which was connected to a water pump that provided continuous water flow to keep the samples at a similar temperature of 27–29°C as on the reef. The aquaria were placed outdoors in full sunlight to mimic the natural high-light environment of *U. lactuca*. Water from the aquarium could not mix with the mineral medium in the incubators. Each incubator received continuous aeration using two Sera Precision Air 550R Plus membrane pumps (Sera GmbH, Heinsberg, Germany). Each week, the incubation solution was renewed. The NIFT responses of N-starved and P-starved *U. lactuca* leaves to the addition of NO_3_^−^, NH_4_^+^ and PO_4_^3−^ were determined every other day for 19 days.

### Field Samples of Macroalgae and Seagrass

Individual leaves of the encrusting fan-leaf alga (*Lobophora variegata* (J.V. Lamouroux) Womersley ex E.C. Oliveira) were collected from 20 m depth on the coral reefs of research sites Buoy 0 and Playa Kalki by means of SCUBA diving. Leaves of turtle grass (*Thalassia testudinum* Banks ex König) were collected from ∼1 m depth at Boka Ascencion, and cut into 1 cm^2^ pieces. All sampled leaves were manually cleaned of epiphytes and detritus. The leaves were kept at a temperature of 27–29°C and shaded during transport to the laboratory using a small cool box containing ambient seawater. NIFT measurements on the fresh *L. variegata* and *T. testudinum* samples commenced directly after transportation from the field sites to the laboratory, within 1–2 h after sampling. For *L. variegata*, we used 36 leaves per nutrient treatment from Playa Kalki and 36 leaves per nutrient treatment from Buoy 0. For *T. testudinum*, we measured the NIFT response of 20 leaves.

To interpret possible differences in NIFT response of *L. variegata* sampled from Buoy 0 and Playa Kalki, we briefly compared the environmental growth conditions at these two research sites. At both sites, we placed a 100 m horizontal transect line on the coral reef at 20 m depth. Benthic cover of hard corals and macroalgae was determined from photographs of 60 randomly placed quadrates (1.5 m^2^) distributed along both sides of this transect line. The photographs were analysed using the computer program Coral Point Count with Excel Extensions (CPCe) [23]. Furthermore, water samples were taken along the horizontal transect at 10 cm above the reef using a 60 ml syringe (n = 14 at Buoy 0, n = 17 at Playa Kalki). Water samples were quickly filtered at the dive site using a 0.22 µm Acrodisc filter and stored in 6 ml polyethylene vials (PerkinElmer, MA, USA) at −20°C until further analysis. Concentrations of NO_3_^−^ [24], NH_4_^+^ [25], and PO_4_^3−^ [26] were analysed at the Royal Netherlands Institute for Sea Research (NIOZ), the Netherlands, using continuous flow analysis via a Quatro auto-analyzer (Seal Analytical, UK).

### Nutrient-Induced Fluorescence Transient (NIFT) Experiments

**Figure 2:**
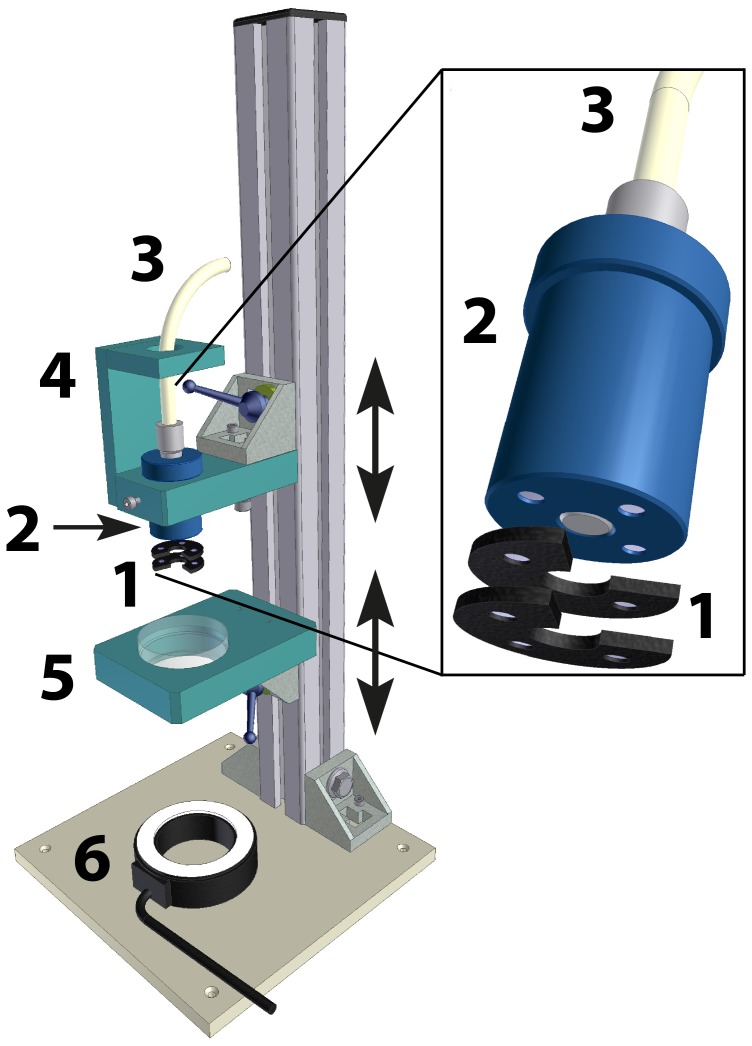
PAM fluoroscope used for NIFT experiments. PAM fluoroscope, consisting of (1) two ¾ magnetic rings for proper sample placement in front of PAM sensor; (2) magnetic PAM sensor head; (3) PAM sensor; (4) adjustable holder for placement of PAM sensor; (5) adjustable Petri dish holder; (6) LED-light with adjustable light intensity.

Changes in variable chlorophyll *a* fluorescence in response to different nutrient additions were measured with a Diving-PAM/B Underwater Fluorometer (Walz Mess- und Regeltechnik, Effeltrich, Germany) using the experimental set-up shown in Fig. 2. Individual *U. lactuca*, *L. variegata*, and *T. testudinum* leaves were placed between two 2 mm thick ¾ round magnetic rings (see insert in Fig. 2) and attached to a magnetic sensor head to ensure that the samples were situated exactly 2 mm in front of the PAM sensor [27]. The sensor head with the attached sample was then placed inside a Ø 54 mm Petri dish containing 15 ml of either enriched seawater (laboratory incubations of *U. lactuca*) or ambient seawater (field samples of *L. variegata* and *T. testudinum*). The use of the ¾ magnetic rings ensured that the nutrient solution always reached the entire leaf surface of the sample on both sides.

Before each NIFT experiment, samples were incubated in the dark for 10 min. Subsequently, at the start of the NIFT experiment, the weak measuring light of the PAM fluorometer was switched on to determine (1) the initial fluorescence (F_0_) and (2) maximum fluorescence following a saturating light pulse (F_m_). Thereafter, samples were exposed to actinic light (PAR, 400–700 nm) of 110 µmol photons m^−2 ^s^−1^ provided by a LED-56 Microscope Ring Light (AmScope Corp., Irvine, CA), to monitor (3) steady-state fluorescence (F_t_), and (4) maximum fluorescence following a saturating light pulse (F′_m_). F_t_ and F′_m_ were measured at 30 s intervals (PAM settings: measuring light = 10, gain = 2, SW = 0.4, SI = 4). After 10 min, a 1.5 ml control solution (with the same nutrient composition as in the incubation glass for *U. lactuca*; with ambient seawater for *L. variegata* and *T. testudinum*) was added to the Petri dish to check whether the addition itself caused a change in fluorescence. After another 5 min, different nutrient solutions were added at 5-min intervals to assess changes in the fluorescence parameters (F_t_ and F′_m_) upon nutrient resupply. A typical NIFT experiment lasted 30 to 60 min in total (including the 10 min of dark incubation).

Nutrient uptake rates of macroalgae and seagrasses are often enhanced when nutrients are supplied in combination with water movement. However, water movement is not desirable during NIFT experiments, as our observations showed that mild movement of the leaves was already sufficient to affect the fluorescence signal. To overcome the limited mass transfer of nutrients across the boundary layer of leaves incubated in stagnant water, we therefore applied relatively high nutrient concentrations in the nutrient additions, ranging from 10 to 250 µM of NO_3_^−^, NH_4_^+^ and PO_4_^3−^. These concentrations are similar to those applied in earlier microalgal studies [15]. In pilot experiments we measured NH_4_ uptake rates and NIFT responses of *U. lactuca* under controlled laboratory conditions at 10, 100 and 200 µM NH_4_ concentrations (unpublished data, J. den Haan), since it is known that NH_4_ can have toxic effects at high concentrations. The results did not show any unusual NIFT responses. Furthermore, NH_4_ uptake rates were not suppressed at the higher NH_4_ levels, and were of similar magnitude as in previous studies with macroalgae [28], [29]. This indicates that the added NH_4_ was not toxic across this concentration range. Our first NIFT experiments, with *U. lactuca*, indicated that a dosage of 100 µM gave the most reliable results. Hence, we chose 100 µM additions of NO_3_^−^, NH_4_^+^ and PO_4_^3−^ for our subsequent NIFT experiments with *L. variegata* and *T. testudinum*.

The fluorescence measurements were used to calculate the quantum yield of photosystem II (Φ_PSII_) according to [30]:
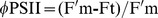
(1)


The quantum yield of photosystem II expresses the fraction of photons absorbed by photosystem II that is used for photosynthetic electron transport. It can thus be interpreted as a measure of photosynthetic efficiency, and is widely used as an index for the physiological status of phototrophic organisms [9], [19], [22], [31], [32].

Non-photochemical quenching (NPQ) was calculated as [22]:
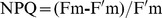
(2)


NPQ is a measure of the photoprotective capacity of phototrophic organisms to dissipate excess energy as heat [22], [33].

### What is a true NIFT response?

**Figure 3:**
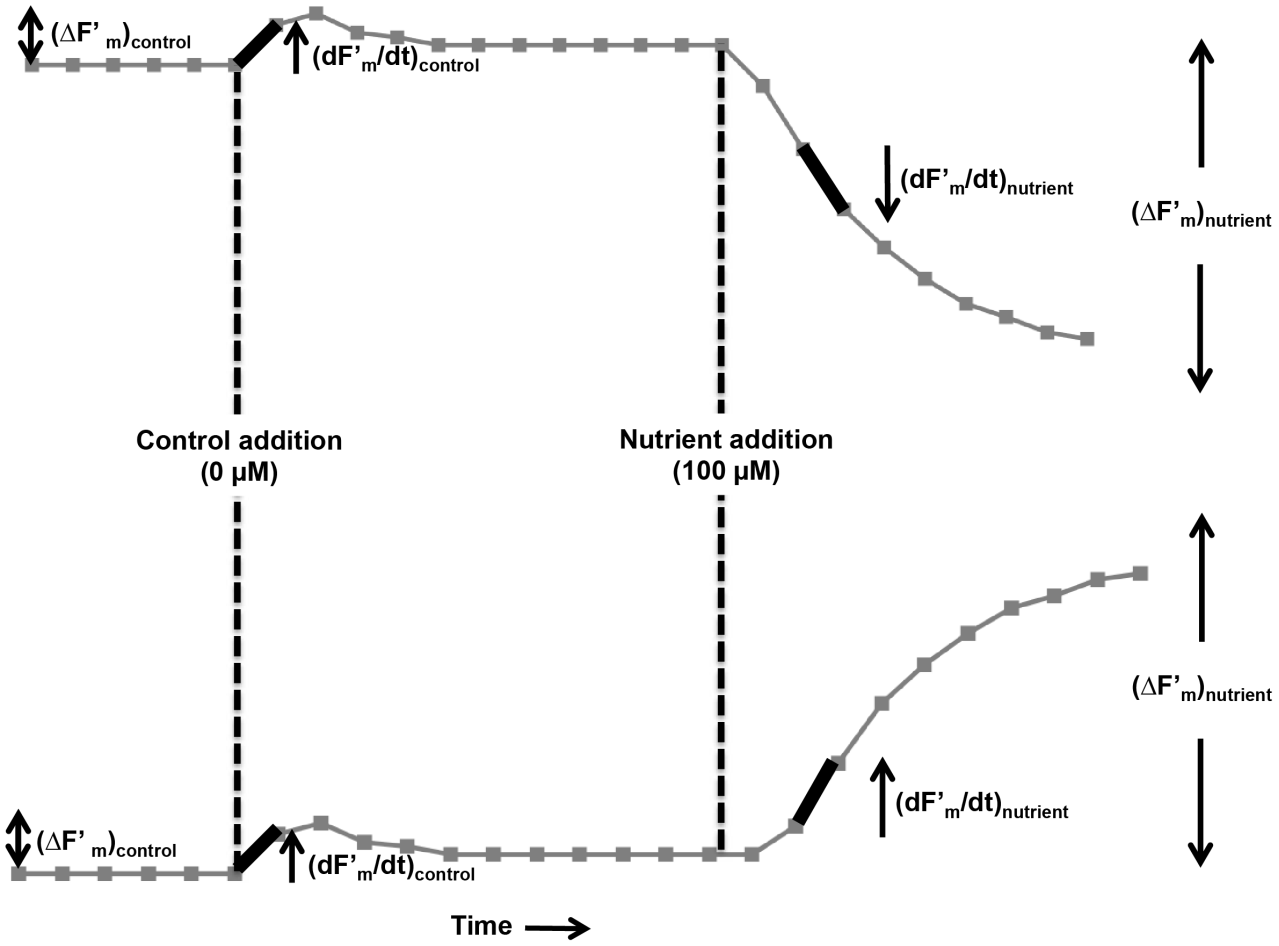
How to determine a NIFT response? Schematic overview of the two criteria used to assess the presence or absence of a NIFT response upon nutrient addition during two possible NIFT reactions. The first criterion compares the rate of change in maximum fluorescence induced by nutrient addition ((dF′_m_/dt)_nutrient_) versus that induced by the control solution ((dF′_m_/dt)_control_). The second criterion compares the total change in maximum fluorescence induced by nutrient addition ((ΔF′_m_)_nutrient_) versus that induced by the control solution ((ΔF′_m_)_control_).



NIFT responses to nutrient addition can sometimes be difficult to interpret, for instance when changes in fluorescence are relatively small or when the control treatment without added nutrient also induces a change in fluorescence [9], [18], [19]. We therefore developed two simple metrics to assess the NIFT response. The first metric (Q_1_) compares the maximum instantaneous rate of change in maximum fluorescence (dF′_m_/dt) induced by the nutrient addition versus that induced by the control solution (Fig. 3):

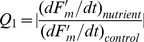
(3)The second metric (Q_2_) compares the total change in maximum fluorescence (ΔF′_m_) induced within 5 min after the nutrient addition versus that induced by the control solution (Fig. 3):
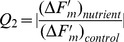
(4)


We judged the NIFT response as real, if the response to nutrient addition was at least twice as large as the response to the control solution (i.e., Q_1_≥2 and/or Q_2_≥2). These criteria are of course somewhat arbitrary. We could have focused on changes in F_t_ or Φ_PSII_ (instead of F′_m_), or we could have set the threshold values of Q_1_ and Q_2_ at another value (instead of 2). However, in 95% of the NIFT experiments with *L. variegata* (n = 108), assessment of the NIFT responses based on these criteria matched our intuitive judgment, which indicated that these criteria provided a useful guideline.

## Results

### Laboratory Incubations of Nutrient-limited *Ulva lactuca*

**Figure 4:**
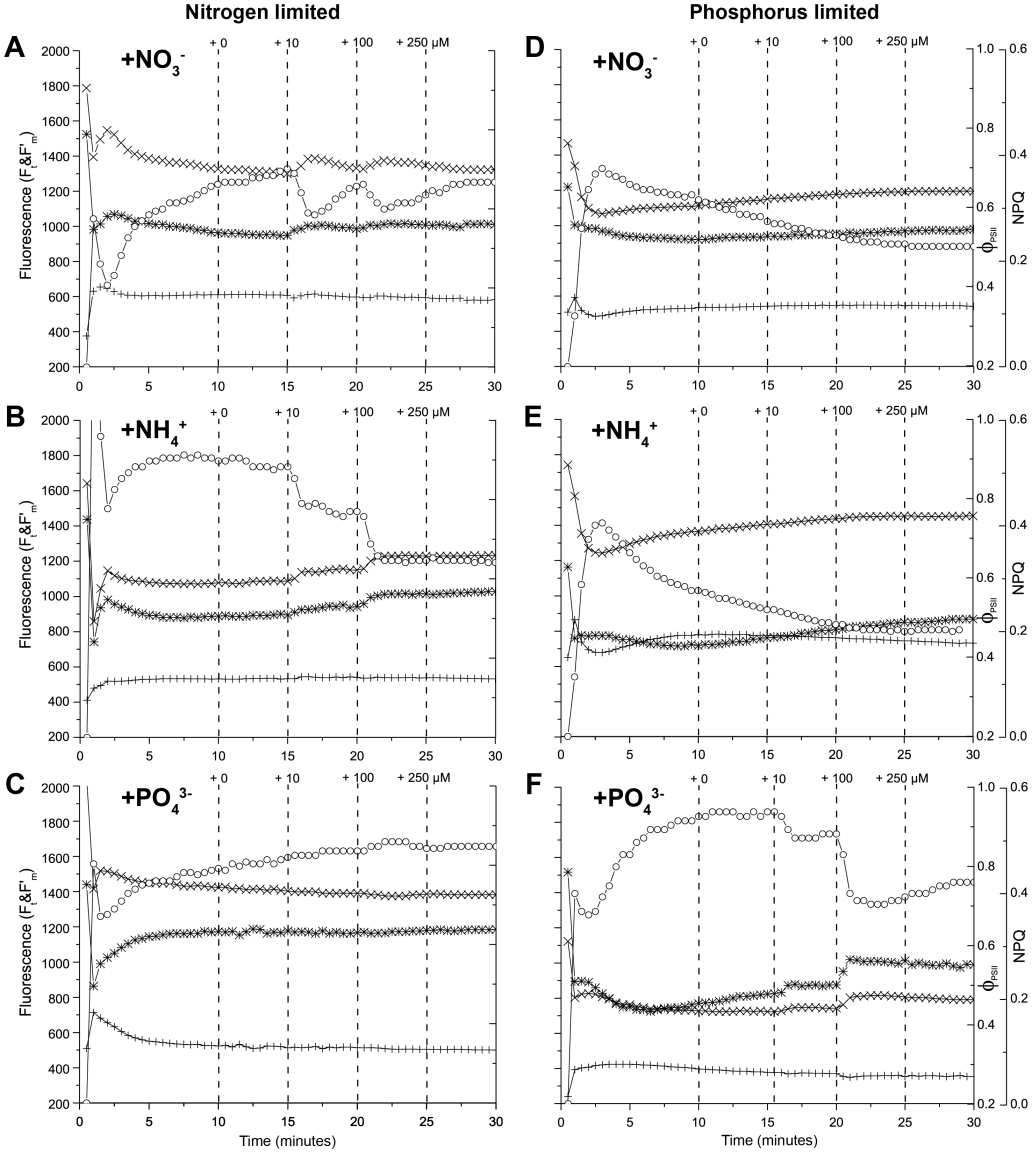
NIFT responses of nutrient-starved *Ulva lactuca*. Examples of the NIFT response of a N-starved *U. lactuca* leaf to (A) NO_3_^−^ addition, (B) NH_4_^+^ addition, and (C) PO_4_^3−^ addition, and a P-starved *U. lactuca* leaf to (D) NO_3_^−^ addition, (E) NH_4_^+^ addition, and (F) PO_4_^3−^ addition. The graphs show the time courses of steady-state fluorescence, F_t_ (+); maximum fluorescence, F′_m_ (×); the quantum yield of photosystem II, Φ_PSII_ (∗); and non-photochemical quenching, NPQ (○). Vertical dashed lines indicate the timing of the control addition (0 µM) and three consecutive nutrient additions (10, 100 and 250 µM).

Fig. 4A shows a typical NIFT response to NO_3_^−^ addition of an *U. lactuca* sample that had been N starved for 11 days. F′_m_ was at its maximum at the first saturating light pulse (i.e., F′_m_ = F_m_ at t = 0), since the sample had previously been dark adapted for 10 minutes. Hence, all PSII reaction centers were ready to carry out photochemistry, while heat dissipation (NPQ) was not yet operational (Eq. 2). After this first light pulse, actinic light was turned on. As a consequence, F′_m_ initially decreased while NPQ increased, indicating that the heat dissipation mechanism was operational from the second light pulse (at t = 0.5 min) onwards. After 20 light pulses (t = 10 min), a control solution with the same nutrient composition as in the incubation glass was added, which did not result in a change in any of the fluorescence variables (F_t_, F′_m_, Φ_PSII_ and NPQ). In contrast, after addition of 10 µM NO_3_^−^ (t = 15 min) and 100 µM NO_3_^−^ (t = 20 min), F′_m_ and Φ_PSII_ increased, whereas NPQ decreased. The addition of 250 µM NO_3_^−^ after 25 min did not result in a response in any of the variables.

Addition of NH_4_^+^ to N-starved *U. lactuca* led to similar results as NO_3_^−^ addition, with an increase of F′_m_ and reduction of NPQ (Fig. 4B). In contrast, addition of PO_4_^3−^ to N-starved *U. lactuca* did not yield a NIFT response in 90% of the cases (n = 10) (Fig. 4C). Conversely, P-starved *U. lactuca* did not respond to the addition of NO_3_^−^ and NH_4_^+^ (n = 8) (Fig. 4D,E), but showed a clear NIFT response to PO_4_^3−^ addition (Fig. 4F).

### Effect of Starvation Period on the NIFT Response

To assess whether the duration of the starvation period affected the results, we investigated the NIFT response during three different time intervals of nutrient starvation (days 1–5, 6–10, and 11–15). We focused on the NIFT response of N-starved *U. lactuca* to NO_3_^−^ and NH_4_^+^ addition, and P-starved *U. lactuca* to PO_4_^3−^ addition, using the same sequence of nutrient additions (10, 100 and 250 µM) as in Fig. 4. In some cases, we did not find a NIFT response at the highest nutrient dosage of 250 µM (see, e.g., Fig. 4A), presumably because the uptake systems were already nutrient-saturated from the earlier addition of 100 µM. Hence, we decided that if the F′_m_ of *U. lactuca* responded to at least one of the three nutrient dosages, this was marked as a positive NIFT response, indicating that *U. lactuca* was indeed N or P limited. Between days 1–5, approximately 50% of the N-starved *U. lactuca* showed a positive NIFT response to NO_3_^−^ and NH_4_^+^ addition, while 33% of the P-starved *U. lactuca* responded to PO_4_^3−^ addition. This indicated that the samples were already nutrient limited from the start of the experiments. The percentage of positive NIFT responses increased up to 60–70% for both N-starved and P-starved leaves of *U. lactuca* after 6–10 days of nutrient starvation. After 11–15 days, the percentage of positive NIFT responses decreased slightly to 47–60%. This coincided with a reduction of Φ_PSII_ to 0.2–0.3 after 15 days of nutrient starvation. For comparison, a healthy nutrient-replete *U. lactuca* leaf has a Φ_PSII_ of 0.6–0.7.

### Field Samples of the Macroalga *Lobophora variegata*

We investigated the NIFT response of *L. variegata* leaves collected from the research sites Playa Kalki and Buoy 0. Playa Kalki is a coral reef ecosystem with ∼25% cover by hard corals and <50% cover by algae (including *L. variegata*) (Table 1). In contrast, Buoy 0 is a more degraded reef ecosystem with only 10% cover by hard corals and almost 60% algal cover. *L. variegata* was nearly twice as abundant at Buoy 0 as at Playa Kalki (Table 1). Concentrations of dissolved NO_3_^−^ and PO_4_^3−^ were significantly higher at Buoy 0 than at Playa Kalki, while the NH_4_^+^ concentration was not significantly different between the two sites (Table 1). The N:P ratio seemed slightly higher at Buoy 0 (16.5∶1) than at Playa Kalki (14.4∶1), indicating that the growth conditions might be relatively more P limited and less N limited at Buoy 0 than at Playa Kalki, but the difference was not significant (Table 1).

**Table 1:** Comparison of environmental characteristics at the research sites Buoy 0 and Playa Kalki.

	Buoy 0	Playa Kalki	U-value	n_1_, n_2_	Significance
Coral cover (%)	10.0±8.3	24.5±14.7	2987.5	60, 60	**P<0.001**
Algal cover (%)	58.8±16.9	48.1±14.7	1140.0	60, 60	**P<0.001**
Cover by *Lobophora variegata* (%)	20.3±15.4	11.8±11.2	1171.5	60, 60	**P<0.001**
Nitrate (µM)	0.261±0.08	0.186±0.23	57.0	14, 17	**P = 0.014**
Ammonium (µM)	0.539±0.39	0.422±0.16	122.0	14, 17	**P = 0.905**
Phosphate (µM)	0.053±0.01	0.042±0.02	55.0	14, 17	**P = 0.011**
N:P ratio (molar)	16.5∶ 1	14.4∶ 1	125.5	14, 17	**P = 0.799**

Comparison of coral cover (± s.d.), total algal cover (including macroalgae, turf algae and benthic cyanobacteria), cover by the macroalga *L. variegata*, dissolved nutrient concentrations and N:P ratios at the coral reef ecosystems of Buoy 0 and Playa Kalki. The data were collected at 20 m depth. Differences between the two research sites were tested with the Mann-Whitney U Test using a significance level of P<0.05; n_1_ and n_2_ indicate the samples sizes at Buoy 0 and Playa Kalki, respectively. Significant P-values are indicated in bold.

**Figure 5:**
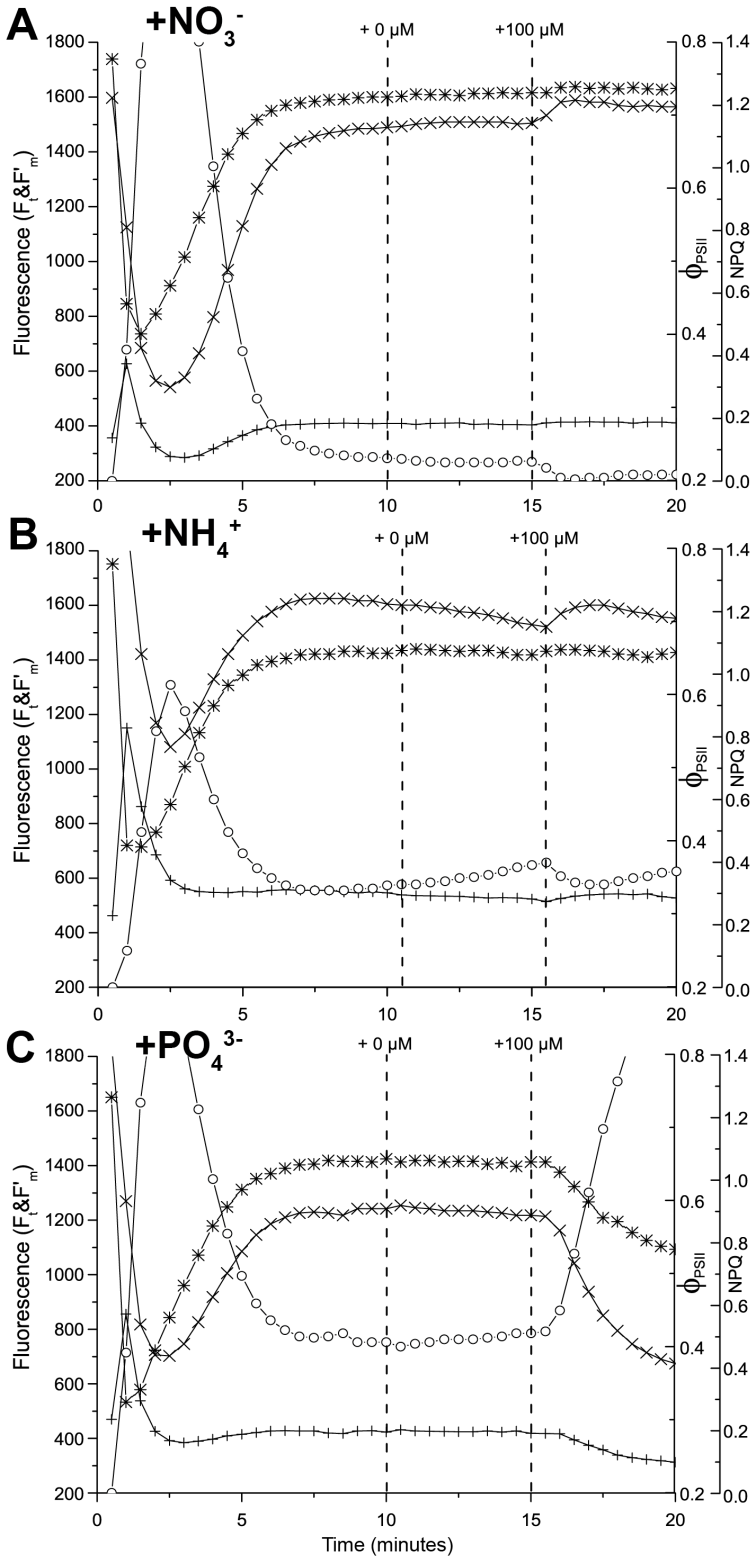
NIFT responses of *Lobophora variegata* collected from the reef. Examples of the NIFT response to (A) NO_3_^−^ addition of a *L. variegata* leaf collected at Playa Kalki, (B) NH_4_^+^ addition of a *L. variegata* leaf collected at Buoy 0, and (C) PO_4_^3−^ addition of a *L. variegata* leaf collected at Buoy 0. The graphs show the time courses of steady-state fluorescence, F_t_ (+); maximum fluorescence, F′_m_ (×); the quantum yield of photosystem II, Φ_PSII_ (∗); and non-photochemical quenching, NPQ (○). Vertical dashed lines indicate the timing of the control addition (0 µM) and different nutrient additions (all at 100 µM).

**Figure 6:**
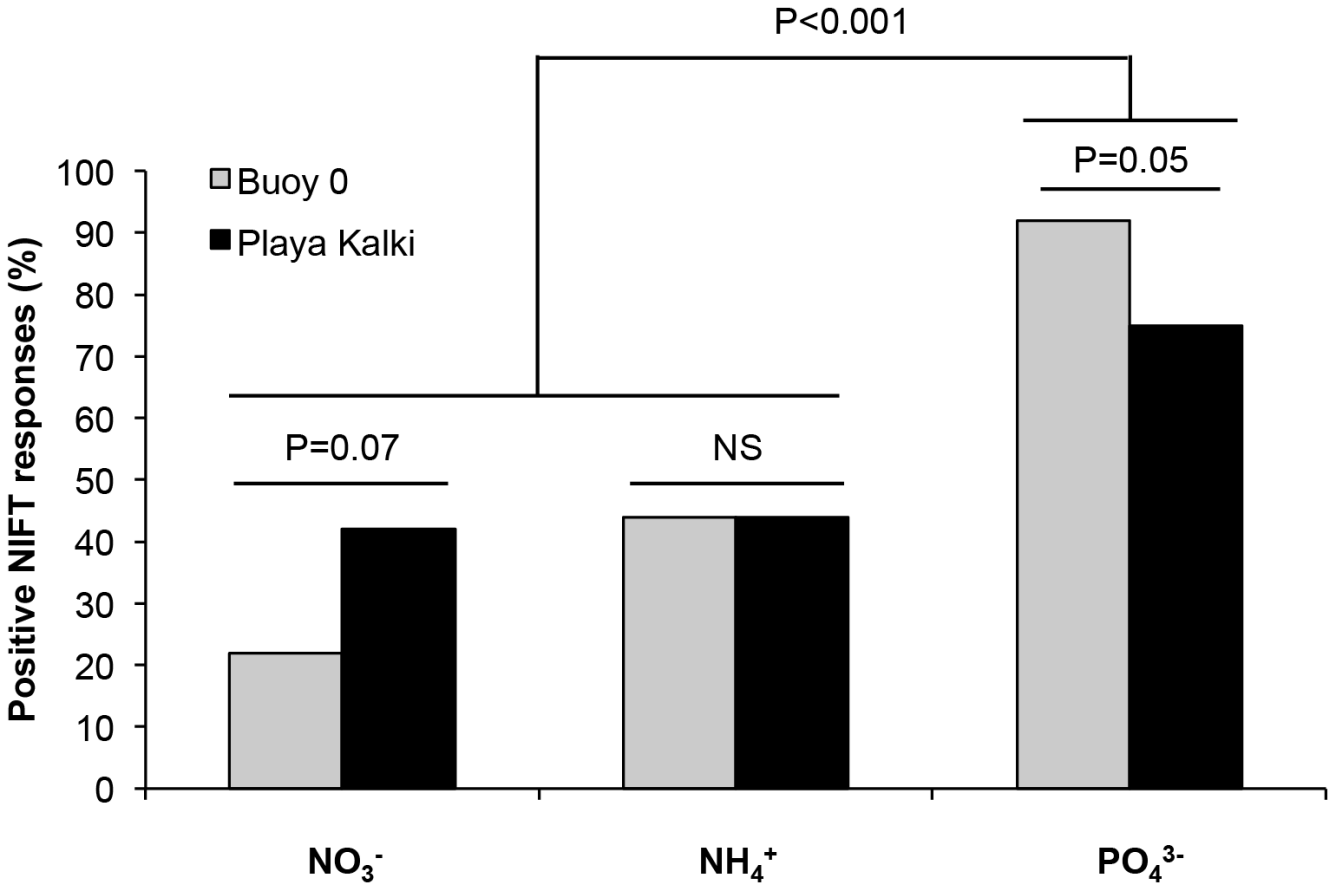
Nutrient limitation of *Lobophora variegata* at two different research sites. Percentage of positive NIFT responses of *L. variegata* leaves, collected from Playa Kalki and Buoy 0, to addition of 100 µM of NO_3_^−^, NH_4_^+^ and PO_4_^3−^. Differences between the two research sites were tested with the Two Proportion Z-test. NS is not significant at P≥0.10; n = 36 per research site and nutrient treatment.

Typical NIFT responses of *L. variegata* to the addition of 100 µM of NO_3_^−^, NH_4_^+^, and PO_4_^3−^ are illustrated in Fig. 5A, B and C, respectively. Interestingly, *L. variegata* showed positive NIFT responses to both N and P additions, although a significantly larger percentage of samples responded to PO_4_^3−^ addition (84%) than to NO_3_^−^ and NH_4_^+^ addition (38%) (Fig. 6; Two Proportion Z-test; Z = 4.5, df = 106, P<0.001). This indicates that *L. variegata* was co-limited by N and P, but with a stronger limitation by P than by N. Moreover, the data suggest that the nutrient limitation pattern was slightly different between the two research sites. That is, although differences between the two sites were only marginally significant, *L. variegata* seemed more strongly limited by PO_4_^3−^ (Two Proportion Z-test; Z = 1.90; df = 70; P = 0.05) and less strongly limited by NO_3_^−^ (Two Proportion Z-test: Z = −1.79; df = 70; P = 0.07) at Buoy 0 than at Playa Kalki (Fig. 6).

When combining all positive NIFT responses of *L. variegata*, NO_3_^−^ addition resulted in an increase in F′_m_ and decrease of NPQ in 87% of all positive NIFT responses. In 13% of the positive NIFT responses, F′_m_ decreased while NPQ increased upon NO_3_^−^ addition. Similar results were obtained for NH_4_^+^ addition, where 72% of the positive NIFT responses showed an increase in F′_m_, and 28% a decrease. Interestingly, the NIFT response of *L. variegata* to PO_4_^3−^ addition showed the opposite pattern, with a decreasing F′_m_ and increasing NPQ in 94% of all positive NIFT responses. An example is shown in Fig. 5C. Conversely, F′_m_ increased while NPQ decreased in only 6% of the positive NIFT responses to PO_4_^3−^ addition.

In a series of extra NIFT experiments we added 100 µM of NO_3_^−^, NH_4_^+^ and PO_4_^3−^ in randomized order at 5 min intervals to the same *L. variegata* sample. This showed that the first nutrient added did not affect the response to the consecutive addition (P = 0.93; Two Proportion Z-test for data Buoy 0 and Playa Kalki combined, n = 61). This can shorten the duration of NIFT experiments substantially. Earlier we investigated each nutrient separately in NIFT experiments of 20 min per nutrient (Fig. 5). Each of these experiments was preceded by 10 min of dark adaptation. Hence, studying NO_3_^−^, NH_4_^+^ and PO_4_^3−^ in three separate NIFT experiments took at least 90 min. Now all three nutrients can be investigated in one run of 10 min dark adaptation plus 30 min of NIFT measurements, reducing the total duration of the experiment to only 40 min.

### Field Samples of the Seagrass *Thalassia testudinum*

**Figure 7:**
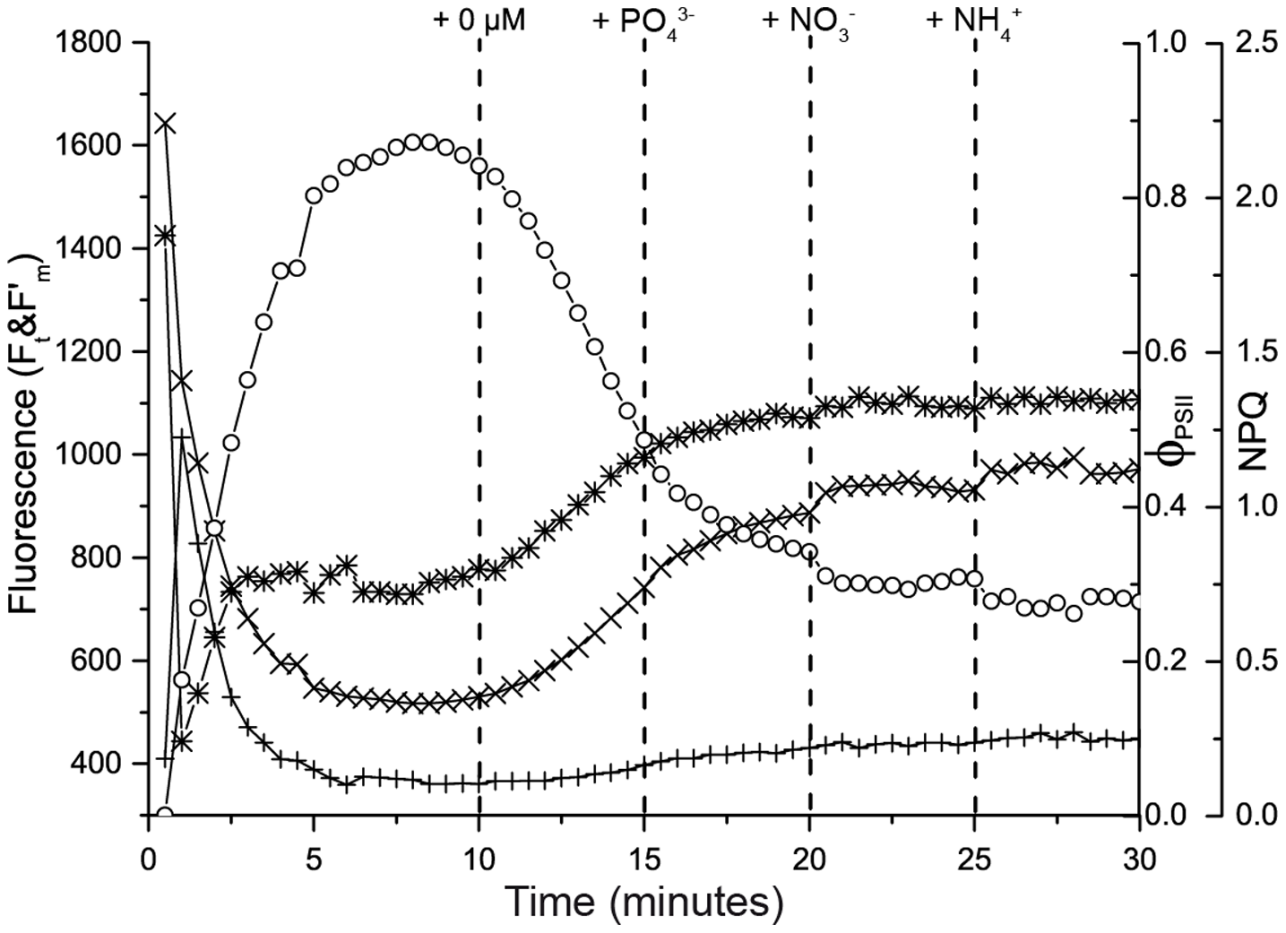
Typical NIFT response of *Thalassia testudinum*. Example of the NIFT response of a *T. testudinum* leaf collected at Boka Ascension. The graph shows the time courses of steady-state fluorescence, F_t_ (+); maximum fluorescence, F′_m_ (×); the quantum yield of photosystem II, Φ_PSII_ (∗); and non-photochemical quenching, NPQ (○). Vertical dashed lines indicate the timing of the control addition (0 µM) and the sequential addition of different nutrients (all at 100 µM).

Fig. 7 shows a typical NIFT response of *T. testudinum* to the sequential addition of 100 µM PO_4_^3−^, NO_3_^−^ and NH_4_^+^ at 5 min intervals. In total, 4 of the 20 *T. testudinum* samples collected from research site Boka Ascencion responded to NO_3_^−^ and/or NH_4_^+^ addition (as in Fig. 7), while 1 sample responded only to PO_4_^3−^ addition. In all these cases, F′_m_ increased while NPQ decreased upon nutrient addition.

## Discussion

### Evaluation of the NIFT Technique

Previous studies have shown that nutrient-induced fluorescent transients (NIFTs) provide an easy and fast means to determine which nutrients limit phytoplankton productivity [15]–[20]. Building upon this existing experience, we aimed to investigate whether NIFT measurements can also assess nutrient limitation in macroalgae and seagrasses. A key ingredient in our approach is the use of a special device that we have called the PAM fluoroscope, which enables exposure of algal thalli and leaves to a series of nutrient additions while keeping these leaves at exactly the same position in front of the PAM sensor. Controlled laboratory experiments with N-starved and P-starved sea lettuce (*U. lactuca*) showed that addition of the limiting nutrient resulted in characteristic changes in chlorophyll *a* fluorescence (F′_m_), while addition of a non-limiting nutrient did not affect the fluorescence signal. Furthermore, we showed that the NIFT technique could detect nutrient limitation of the macroalga *L. variegata* and the seagrass *T. testudinum* directly after they were collected from the field. Hence, our results demonstrate that the NIFT technique can be successfully applied to macroalgae and seagrass, important representatives of the benthic primary producers inhabiting many coastal waters, coral reefs and shallow lakes.

Surprisingly, even during controlled nutrient starvation in the laboratory, the percentage of positive NIFT responses in *U. lactuca* never exceeded 70%. That is, even under stringent limitation, approximately one third of the *U. lactuca* leaves did not show a NIFT response. This contrasts with phytoplankton studies, where laboratory experiments have shown positive NIFT responses in up to 100% of the assays [19]. In our field samples, the maximum percentage of positive NIFT responses was 92% for *L. variegata* but only 25% for *T. testudinum*. The low percentage of positive NIFT responses for *T. testudinum* may indicate that either this species is not very responsive to NIFT measurements, or that it was not strongly nutrient limited at its sampling site in the bay of Boka Ascencion. In contrast to macroalgae, seagrasses like *T. testudinum* can also extract nutrients from the sediment through their root system [34], [35]. Hence, they may be less subjected to nutrient limitation than macroalgae that acquire their nutrients only from the surrounding water column. Further studies comparing nutrient limitation in macroalgae and seagrasses will be required to investigate this hypothesis in more detail. All in all, these results indicate that studies of nutrient limitation in macroalgae and seagrasses using the NIFT technique should always sample a sufficient number of leaves (say, at least 10–20 leaves) to obtain reliable results.

### The Nature of the NIFT Response

A somewhat naive but straightforward explanation for nutrient-induced changes in fluorescence would be that enhanced nutrient assimilation increases the demand for ATP and NADPH. This relieves pressure on photosynthetic electron transport, and, hence, one would expect a decrease in chlorophyll fluorescence. However, our results show that fluorescence can either increase or decrease upon nutrient addition, depending on the nutrient being added and the species being studied. For instance, we found that maximum fluorescence (F′_m_) of *U. lactuca*, *L. variegata*, and *T. testudinum* increased upon NO_3_^−^ addition, while non-photochemical quenching (NPQ) decreased. Similar variation in the NIFT response has also been observed in previous studies with microalgae [15]. An increase in chlorophyll fluorescence and drop in NPQ upon NO_3_^−^ addition was reported for the unicellular green alga *Dunaliella tertiolecta* [36], but another green alga, *Chlorella emersonii*, showed the opposite response [37]. NO_3_^−^ uptake and assimilation requires both ATP and NADPH. Shelly et al. [15] therefore hypothesized that the rise in fluorescence and drop in NPQ might be explained by state transitions between PSI and PSII. State transitions are rapid physiological adaptation mechanisms that adjust the way absorbed light is distributed between the two photosystems. A state transition from State 2 to State 1 will increase the contribution of PSII, and hence linear electron transport to produce both ATP and the required reduction equivalents [15]. Since nearly all chlorophyll fluorescence comes from PSII, such a state transition would increase the fluorescence signal.

Addition of NH_4_^+^ to N-limited *Dunaliella tertiolecta* and *Chlorella emersonii* resulted in an initial rise in fluorescence, which subsequently dropped sharply, before recovering to a new steady state [19], [36], [37]. Conversely, NH_4_^+^ addition to N-limited cultures of the green alga *Monoraphidium minutum* (formerly known as *Selenastrum minutum*) and the cyanobacterium *Oscillatoria* sp. showed an initial drop in fluorescence, followed by an increase towards a new steady state [16], [19]. Such initial oscillations in fluorescence signal were not observed in our experiments with *U. lactuca*, *L. variegata*, and *T. testudinum*, where the fluorescence increased monotonically upon NH_4_^+^ addition.

Upon PO_4_^3−^ addition, a drop in fluorescence is described as the most common NIFT response in P-limited microalgae [15]. The mechanism of a NIFT response to PO_4_^3−^ addition has been studied in P-limited *D. tertiolecta* [20]. The drop in fluorescence appeared to be caused by (1) a state transition from State 1 to State 2, which leads to higher cyclic electron flow around PSI to meet the higher ATP demand for P uptake, and (2) increased non-photochemical quenching by an enhanced xanthophyll cycle activity, which dissipates excess light energy as a protective mechanism to avoid photodamage to the photosynthetic machinery [20]. Yet, we observed a drop in fluorescence upon PO_4_^3−^ addition only in *L. variegata,* while *U. lactuca* and *T. testudinum* showed a rise in fluorescence upon PO_4_^3−^ addition. Clearly, the exact underlying mechanisms explaining the variation in NIFT response between different species and nutrient additions are yet to be further determined [15].

Earlier studies with microalgae indicated that the magnitude of the NIFT response increases with the severity of nutrient limitation [19], [36]. For instance, Holland et al. [19] sampled natural phytoplankton populations from several Australian waters, and did not observe any positive NIFT responses on the day of collection. Positive NIFT responses appeared only after the samples had been exposed to several days of nutrient starvation under controlled laboratory conditions. This contrasts with our findings, where *U. lactuca*, *L. variegata* and to a somewhat lesser extent also *T. testudinum* all showed positive NIFT responses on the day of collection. Moreover, freshly collected *U. lactuca* showed relatively mild changes in the percentage of positive NIFT responses during the subsequent two weeks of nutrient starvation in controlled laboratory incubations. This indicates that *U. lactuca*, and probably also the other two species that we investigated, were already strongly nutrient limited prior to sampling, i.e., in their natural habitat.

### Co-limitation by Nitrogen and Phosphorus

Our results indicate that at least part of the natural population of *L. variegata* was co-limited by nitrogen and phosphorus. Previous NIFT studies with phytoplankton grown under controlled nutrient conditions have shown that addition of the limiting nutrient produces a positive NIFT response, whereas addition of non-limiting nutrients generally does not cause a change in fluorescence [9]. The same pattern was observed in our laboratory incubations with the macroalga *U. lactuca*, where addition of nitrogen to N-starved leaves and addition of phosphorus to P-starved leaves resulted in a positive NIFT response, while addition of non-limiting nutrients did not affect the fluorescence signal. Hence, the observation that freshly collected leaves of *L. variegata* showed positive NIFT responses to both nitrogen and phosphorus addition points at co-limitation by these two nutrients. Co-limitation by N and P is consistent with the low concentrations of dissolved inorganic nitrogen and phosphorus, at a N:P ratio close to the Redfield ratio of 16∶1, measured in ambient seawater at both research stations Playa Kalki and Buoy 0 (Table 1). Interestingly, the NIFT data even picked up a subtle difference in N:P ratios between the two research sites, as *L. variegata* was somewhat more P limited and less N limited at Buoy 0 than at Playa Kalki.

Co-limitation by N and P has also been observed for several macroalgal species of the Great Barrier Reef, Australia, including *Sargassum baccularia*, *Hydroclathrus clathratus*, *Turbinaria ornata*, and *Padina tenuis* [38]–[40], where the addition of short-term N and P pulses resulted in increased primary production and/or incorporation of these nutrients into their thalli as temporary storage to sustain growth during periods of low nutrient availability. Since co-limitation has not been investigated in earlier NIFT studies [15], our study seems to be the first to demonstrate that co-limitation by two nutrients can be detected with NIFT measurements.

### Perspectives for Application

Our results show that the NIFT technique can be successfully applied to macroalgae and seagrass. The method is relatively fast and straightforward, and provides important information on the nutrients limiting the photosynthetic rates of primary producers. For instance, the macroalga *L. variegata* is one of the key algal species involved in large-scale shifts from coral to macroalgal dominance in coral reef ecosystems across the globe, including the Caribbean Sea [41], [42] and the Great Barrier Reef [43], [44]. Our finding that *L. variegata* is co-limited by nitrogen and phosphorus on the coral reefs of Curaçao, and reaches higher abundances in more nutrient-rich waters near urbanized areas (Table 1), indicates that eutrophication of these coastal waters is likely to enhance the capacity of this algal species to overgrow coral reefs. Further expansion of *L. variegata* and other algal species involved in the degradation of coral reef ecosystems may be curtailed by reductions in nitrogen and phosphorus loads from terrestrial sources, for instance by more extensive wastewater treatment. These results illustrate that use of the NIFT response to assess the nutrient status of primary producers can serve as a valuable tool in coastal management. While we worked on macroalgae and seagrass in tropical marine ecosystems, we foresee a wider application of this method to other benthic algae and submersed aquatic plants in other marine and freshwater habitats.
